# Evaluating the Efficiency of Enhanced Coagulation for Nanoplastics Removal Using Flow Cytometry

**DOI:** 10.1021/acsestwater.5c00219

**Published:** 2025-06-11

**Authors:** Elorm Obotey Ezugbe, Samuel Benjamin Rutten, Bianca de Vries-Onclin, R. Martijn Wagterveld, Wiebe de Vos, Saskia Lindhoud

**Affiliations:** 1 Membrane Science and Technology, 3230 University of Twente, Drienerlolaan 5, Enschede 7522 NB, The Netherlands; 2 Wetsus, European Centre of Excellence for Sustainable Water Technology, Oostergoweg 9, Leeuwarden 8911 MA, The Netherlands; 3 Department of Molecules & Materials, 3230 University of Twente, Enschede 7522NB, The Netherlands

**Keywords:** nanoplastics, coagulation, flocculation, flow cytometry, synopsis

## Abstract

Efficient removal and accurate quantification of nanoplastics in conventional water treatment systems remain closely interconnected challenges. Optimizing removal processes requires robust detection techniques, and the lack of reliable quantification methods hinders process development and validation. In this study, we investigated enhanced coagulation-flocculation techniques for removing fluorescent PS-OSO_3_^–^ nanoplastics of different sizes and concentrations from water. Removal efficiency was assessed using flow cytometry (FCM) and compared to a turbidity-based assessment. Coagulation-flocculation was achieved with Fe^3+^ concentrations ranging from 2 to 30 mg/L and varying slow mixing speeds of 100, 50, and 25 rpm. The results demonstrate that FCM quantifies nanoplastics more reliably and accurately than turbidity measurements at lower nanoplastic concentrations. Enhanced coagulation was achieved at a slow mixing speed of 25 rpm (*G* = 14 s^–1^). Among the factors studied, particle size emerged as the most significant factor influencing the coagulation-flocculation performance. Additionally, sweep coagulation was predominant at low nanoplastic concentrations, while a combination of sweep coagulation and charge neutralization was observed at higher concentrations. These findings provide critical insights into developing effective nanoplastic removal strategies through interconnected advancements in the detection and treatment optimization of conventional water treatment systems.

## Graphical abstract

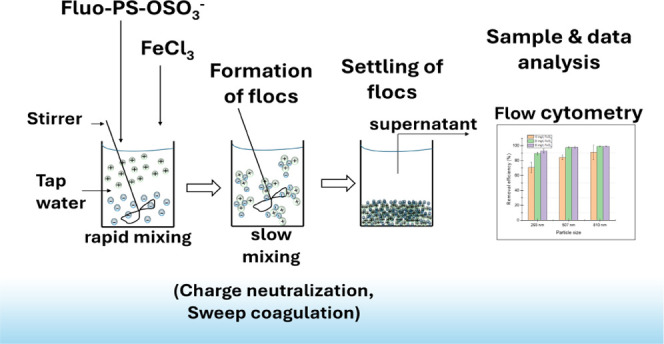

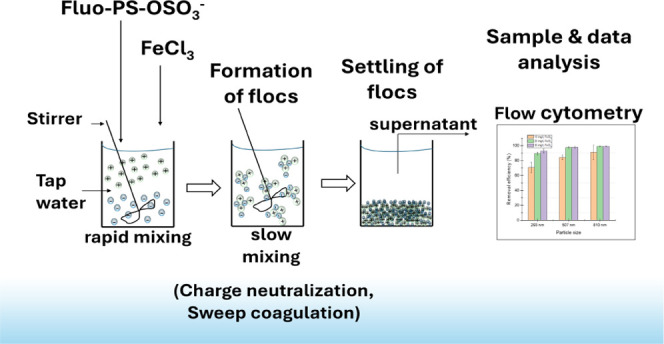

## 1 Introduction

Nanoplastics (NPs) are a heterogeneous group of plastic particulates within the size range of 1 nm–1 μm.^1^ These particles are manufactured for commercial purposes^2,3^ or formed through the breakdown of larger-sized plastic materials.^4–6^ NPs possess complex physicochemical properties such as varying chemical compositions, shapes, and varying degrees of surface charge and aging. Additionally, these particles have a large surface area-to-volume ratio and they possess the ability to form stable colloids in water.^1,7,8^ Due to these inherent properties, we come to two main challenges for NPs: (i) Their accurate detection is difficult due to their small size and similarities in chemical makeup to naturally occurring organics such as proteins and humic acids^9^ and (ii) the removal of NPs from water during treatment is a challenge, especially for conventional water treatment facilities which are not optimized for this purpose.^7,10^ Consequently, NPs are persistent in the water cycle, often at unknown concentrations, posing risks to biota and human health.^11,12^ It is important to note that these two challenges are interconnected, as water treatment processes can only be optimized for NP removal when effective NP detection methods are developed. Therefore, efforts are required to enable accurate detection of NPs so that conventional water treatment processes can be optimized to allow for the successful and cost-effective removal of NPs.

Over the past couple of years, efforts toward NP removal from water using conventional treatment processes have focused on coagulation and flocculation processes.^13–17^ These processes form key parts of the existing water and wastewater treatment trains, and their efficiency significantly affects the efficiency of the other treatment methods in the train, thus affecting the eventual water quality and the overall cost of treatment.^18^ During coagulation, chemicals, known as coagulants, are introduced into water to destabilize suspended and colloidal particles in the water to form microflocs.^19^ These microflocs are then induced to aggregate by making contact with each other, often facilitated by mechanical agitation, and grow into larger agglomerates. This is known as flocculation.^19,20^ One major factor that affects the coagulation and flocculation process is the size of the particles in suspensions.^21^ The smaller the particle, the more challenging their destabilization, aggregation, and subsequent removal from water.^21,22^ Thus, the efficiency of the coagulation and flocculation process is dictated by how easily fine particles in aqueous suspensions are induced to aggregate. However, research conducted by Zhang and coworkers^3,13,23–26^ has shown that conventional coagulation and flocculation processes lack the efficacy to remove fine particles (<10 μm) from water. This existing challenge has been aggravated by the influx of NPs in our water systems. Improvements are therefore needed to induce rapid aggregation of NPs during coagulation and flocculation, to enhance their removal from water.

In water treatment facilities, turbidity measurements represent the most widely used technique to assess the effectiveness of coagulation and flocculation for particulate removal.^27,28^ Previous studies used turbidity measurements to monitor plastic particle removal from water during coagulation and flocculation.^29–31^ However, turbidity measurements are limited in a variety of ways and have been proven to be a weak indicator of particulates in water.^32^ First, the correlation between turbidity data and suspended particle concentration is weak due to the complex interaction of light with suspended particles in the water.^33^ Hence, empirical approaches are adopted to determine the concentrations of suspended particles from turbidity measurements. However, these methods are associated with wide margins of error, making them less applicable to quantify particulate removal.^33^ Second, for complex polydisperse systems, turbidity measurements do not give accurate information about particle size distribution, as large particles will dominate the light scattering behavior.^27,34^ Finally, at low particle concentrations, turbidity becomes less sensitive to changes in the system.^32,35^ To accurately evaluate the extent of nanoparticle (NP) pollution in water and understand the kinetics of NP aggregation, it is essential to determine the particle number concentration (particles/L).^21,36^ Other techniques that have been widely combined with coagulation and flocculation processes for NPs quantification include individual counting of particles using a microscope^37,38^ and gravimetric analyses.^25^ While these are helpful techniques, they can be cumbersome, time-consuming, and prone to errors.^39,40^

In recent times, the use of flow cytometry (FCM) in NP analysis has gained some attention. FCM is a high-resolution, single-particle analytical technique that operates based on the detection of fluorescence signals emitted by a particle as it passes a laser beam in a fluid stream.^41,42^ Studies conducted by Bianco and coworkers^43–45^ explored the possibility of quantifying environmental microplastic (MP) samples using FCM, by staining the samples with Nile Red (NR) dye. The authors concluded that FCM provides a great opportunity for micro- and nanoplastics quantification. Again, in a study conducted by Rajala,^46^ FCM was combined with turbidity measurements and COD to quantify the removal of polystyrene MPs (1 μm fluorescent PS and 6.3 μm nonfluorescent PS) from secondary municipal wastewater after coagulation and flocculation. The authors spiked the wastewater samples with the plastic beads (0.1 mg/L–1.82 × 10^8^ MP/L for 1 μm particles and 6.7 mg/L–5.0 × 10^7^ MP/L for 6.3 μm particles) and compared the removal efficiency of three different coagulants (ferric chloride, polyaluminum chloride, and polyamine). The authors noted that FCM was highly efficient in quantifying the MPs. However, much is still left to be determined when particle size reduces significantly to the nanoscale and particle concentration increases. Particles within the nanoscale have been found to behave quite differently from larger size particles (>1 μm) in terms of their interaction with light, interaction with other particles, transport through water, and their chemical behavior.^8,47^

In this study, we investigated the removal of fluorescent model polystyrene nanoplastics (PS NPs) from water during enhanced coagulation and flocculation. Fluorescent PS NPs beads served as a good model system to study and monitor NPs removal with FCM during coagulation and flocculation. Polystyrene nanoparticles have been widely used in NPs research due to their toxicity, abundance in the environment, and availability in a wide range of sizes, having interfacial and colloidal properties that are relevant to nanoplastics research.^48–51^ We explored the use of the fluorescence-based flow cytometry analysis to accurately quantify and monitor the removal of fluorescent model nanoplastics from water during coagulation/flocculation. Additionally, we compared the efficacy of FCM to accurately quantify NP removal with commonly used turbidity measurements. Finally, we investigated the effects of parameters such as particle size, coagulant dose, and slow mixing speed on NP removal and enhancement of the coagulation/flocculation process.

## 2 Materials and Methods

### 2.1 Nanoplastics: Polystyrene beads

Aqueous suspensions of fluorescent monodisperse polystyrene particles with sulfate end groups (PS-OSO3-) (2.5 wt %, density 1.05 g/cm^3^) were obtained from MicroParticles GmbH (Berlin, Germany) and used without further modification. The spherical, hydrophobic particles were obtained in three sizes: 293 (green), 507 (red), and 810 nm (green). Stock solutions were prepared by diluting the original suspension with Milli-Q water (*R* = 18.2 MΩ/cm, TOC < 5pbb), (Milli-Q Advantage A10, United Kingdom) and stirred with a magnetic stirrer (Heidolph instruments, Germany) at 500 rpm for 30 min to ensure that the particles were well dispersed. Stock solutions were stored in a cool, dark place at 4.0 °C.

### 2.2 PS NP Characterization

To measure the zeta potential and hydrodynamic diameter of the PS NPs, nanoparticle tracking analysis (NTA) was conducted using a Nanosight NS500 (Malvern Instruments Ltd., UK) at 25 °C. The equipment utilizes a laser to illuminate particles in suspension while tracking their Brownian motion with a camera. The measurements were conducted in the fluorescent mode, using a green laser (532 nm) for the green particles (size = 293 and 810 nm) and a red laser (642 nm) for the red particles (size = 507 nm). Samples for zeta potential measurements were prepared using Milli-Q water; 2 mg/L of each particle was prepared and used for the analysis. Prior to each analysis, the fluidics of the system was flushed using Milli-Q. This was followed by priming of the fluidics, also with Milli-Q, allowing the sample chamber to be uniformly filled without any air pockets. Afterward, 0.6–1 mL of sample was then loaded for analysis. Zeta potential measurement was achieved through a combination of drift and Brownian motion under the application of an electrical potential.

In order to visualize and better understand the mechanism of aggregation of the NPs, scanning electron microscopy (SEM) (JEOL, JSM-6480LV, Japan) analysis was performed for samples before and after coagulation. To obtain SEM samples before coagulation, small volumes of 2 mg NP/L of NP suspension were dried overnight on a 0.2 μm polycarbonate (PC) membrane (Merck Millipore, Netherlands). After coagulation, flocculation, and settling, SEM samples were obtained by filtering 5 mL of the sediments through a 0.2 μm PC membrane and dried in an oven at 35 °C for at least 24 h before analysis.

### 2.3 FeCl_3_ and Working Solution

A 1 M FeCl_3_ (Thermo Scientific, The Netherlands) stock solution was prepared and diluted to the desired concentrations varying from 2 to 30 mg Fe^3+^/L. Samples were prepared by spiking tap water with fluorescent 810 nm PS NPs to three different concentrations: 0.2, 2, and 20 mg/L. These concentrations were chosen to cover a wide range of NP concentrations within surface water, groundwater, and wastewater.^52,53^ Additionally, within this range, it was possible to test and compare the viability of the two NP quantification techniques, FCM and turbidity measurements, used in this study. To assess the influence of particle size on removal efficiency, 2 mg/L of each particle (293, 507, and 810 nm) was prepared, and the coagulant dose was varied from 10 to 30 mg Fe^3+^/L. The total working volume during all experiments was maintained at 150 mL. The composition of the tap water can be found in the Supporting Information (SI Table S1).

To test the applicability of FCM for nanoplastics quantification in environmental samples, experiments were conducted by spiking real surface water from the Potmarge, a river in Leeuwarden, The Netherlands (53° 11′ 40.16″ N, 5° 48′ 27.69″ E) with three different concentrations of NPs (0.2, 2, and 20 mg/L of 810 nm PS NPs). Samples were first filtered using a 12–15 μm filter paper (Whatman, Merck, The Netherlands) (Figure S1) before experiments were conducted.

### 2.4 Enhanced Coagulation/Flocculation Experiments

Coagulation/flocculation experiments were conducted in batch mode using a six-paddle jar tester (Velp Scientifica Inc., United States) modified to hold a maximum sample volume of 250 mL. The equipment allows for the simultaneous experimentation of 6 different samples with up to six different conditions. For the enhanced coagulation process, rapid mixing was maintained at 300 rpm (*G*-value = 632 s^–1^) for 1 min, and slow mixing speed was varied at 100, 50, and 25 rpm (*G*-values = 125, 40, and 14 s^–1^, respectively) for 15 min. After coagulation/flocculation, samples were left to settle for 30 min. Immediately after the settling time had elapsed, supernatant samples were drawn 1 cm below the water surface for analysis. Five mL samples were drawn into an Eppendorf tube for further processing for FCM analysis. For turbidity measurements, 30 mL samples were collected directly into clean glass vials using a 10 mL pipet and analyzed immediately. Vials were kept free from debris by using dust-free tissue. All experiments were conducted in triplicate.

### 2.5 Analytical Methods

#### 2.5.1 Flow Cytometer Analysis

Particle concentration of the fluorescent PS NPs was determined using a Cytoflex flow cytometer (Beckman Coulter, United States). The instrument was equipped with a violet laser (405 nm), blue laser (488 nm), and red laser (638 nm). For data acquisition and analysis, CytExpert software (version 2.5) was used.

Samples for FCM analysis were prepared by first vortexing 5 mL of the supernatant from the coagulation process to ensure homogeneity at 2500 rpm using a Heidolph REAX top vortex (Heidolph Instruments, Germany) for approximately 5 to 10 s. Then, a minimum of 250 μL of sample was loaded in each well of a 96-well plate. Analysis was conducted at a sample flow rate of 10 μL/min, with a maximum particle count of 10,000 particles per well. The maximum time for analysis was set to 2 min per sample.

All samples were excited using the blue laser for the fluorescence signal and with violet side scatter (V-SCC) for the side scatter, which is more precise in detecting smaller particles compared with the traditional side scatter (SCC) that uses a blue laser. Fluorescence detection was performed with 610/20 and 525/40 nm band filters for the red and green particles, respectively. The equipment analyses samples and generates dot plots of fluorescence intensity (*x*-axis) versus the degree of scattering (V-SCC) (*y*-axis), which indicates the complexity of the studied particles. Each dot represents a singular particle, as shown in Figure 1.

**Figure 1:**
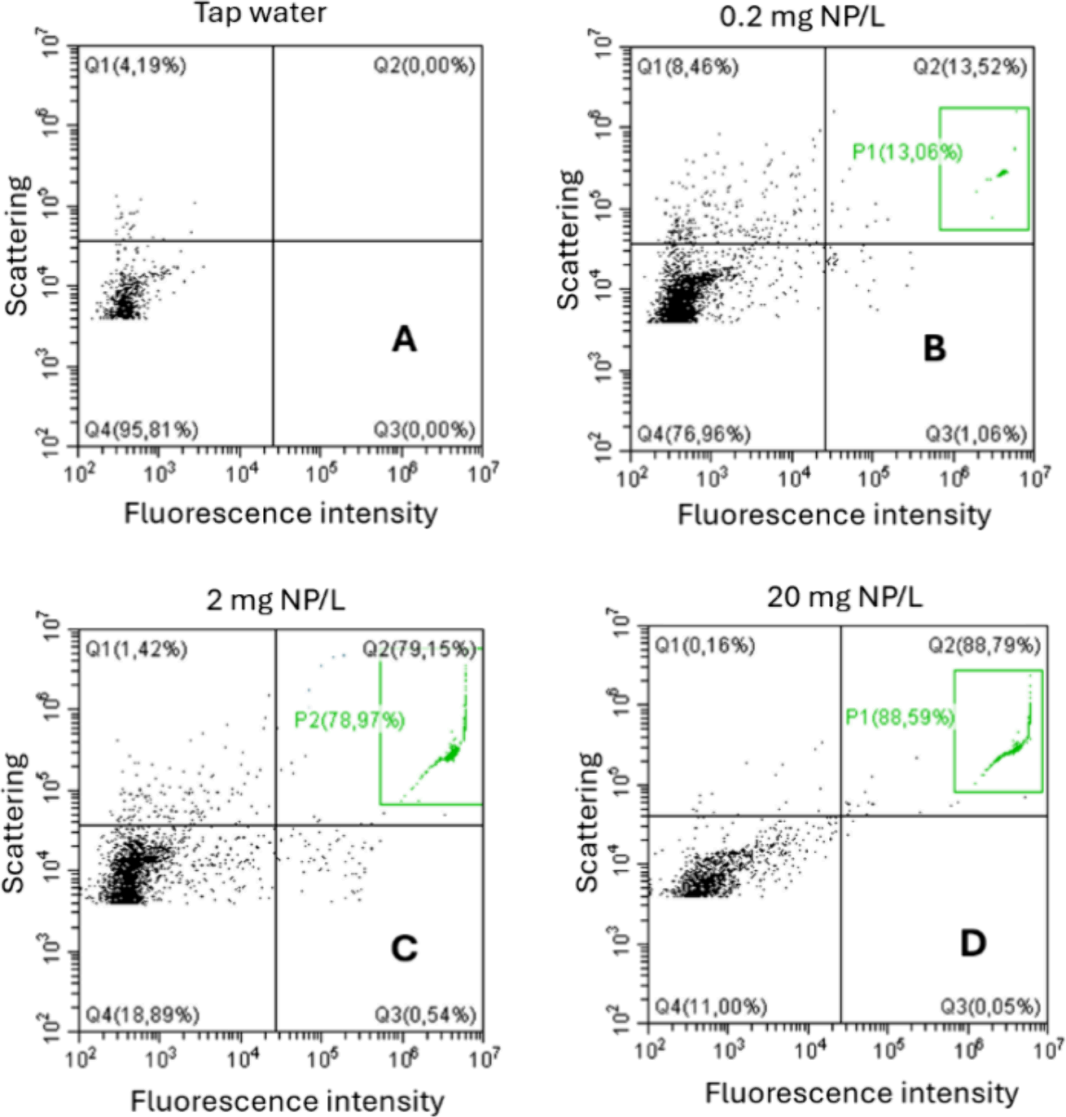
Dot plots of 810 nm PS NPs generated from the FCM. (A) Raw tap water; (B) 0.2 mg/L NP; (C) 2 mg/L; (D) 20 mg/L. *X*-axis = fluorescence intensity after being filtered through B525–H emission filter; *Y*-axis = degree of scattering violet light (VSSC-H @ 405 nm) by particles.

To ensure the quality and accuracy of the measurements, quality control checks were performed before the analysis. This involved the use of standard quality control fluorospheres (CytoFLEX ready-to-use daily QC fluorospheres). The fluorospheres have a mean diameter of 3.0–3.4 μm and were expected to generate a minimum flow rate of 100 events (particles)/second during quality control checks as verification of the alignment of the fluidics. In addition, negative controls (Milli-Q) were included in each analysis (first 4 wells on the 96-well plate: A1 - A4). To avoid carryover from one sample to the other, Milli-Q water was run between samples, as shown in Figure S2.

Furthermore, to establish the optimum detection range for the instrument, the lower and upper limits of detection were determined prior to analysis. The lower limit was found to be 10^4^ particles/L while the upper limit was found to be 10^10^ particles/L. This was determined through gradient dilution of the PS NPs used in this study. The optimum concentration range for the FCM was determined to be 10^7^–10^9^ particles/L, which was also observed by ref 54. Within this range, the correlation between spiked particles and detected fluorescent signals was established.

#### 2.5.2 Turbidity Measurements

Turbidity measurements were conducted using the HACH 2100N laboratory turbidimeter (HACH, United Kingdom). The equipment was fitted with a tungsten lamp, which emits a broad spectrum of light (350–700 nm) and can measure turbidity within the range of 0.1–1000 NTU. To ensure that the quality of measurements is maintained, regular calibration of the equipment was done with StabCal turbidity calibration standards (<0.1–1000 NTU) provided by the manufacturer. Samples were measured by inserting the glass vial into the turbidimeter, waiting for 20–30 s for the reading to stabilize, and taking the value output from the equipment. All measurements were done at least two times.

The removal efficiency (*R*) of nanoplastics and turbidity was calculated as follows^46^

$$R=\frac{C_{i}-C_{f}}{C_{i}}\times 100$$ 1

where *C*_*i*_ is the initial particle concentration or turbidity of samples and *C*_*f*_ is the final particle concentration or turbidity of samples. The removal efficiencies presented are the mean values with their corresponding standard deviations.

## 3 Results and Discussion

### 3.1 Nanoplastics Characterization

The zeta potential and average hydrodynamic diameter of the studied particles were determined by nanoparticle tracking analysis (NTA). As expected, all particles exhibited negative zeta potentials due to the negatively charged sulfate groups on the surface of the polystyrene nanoplastics (Table 1). The smallest particles showed a zeta potential of −28.9 ± 4.3 mV, sufficient to maintain stability in suspension. This is evident in the SEM image (Table 1), where particles appear to be spaced out but form small aggregates. Such behavior is consistent with the zeta potential range of −20 to −30 mV, indicative of moderate stability and the potential for microaggregate formation.^55^ The 507 and 810 nm particles displayed a zeta potential of −35.8 ± 7.9 and −35.1 ± 10.5 mV, respectively, exceeding the threshold of −30 mV, which suggests high stability in suspension.^55^ SEM images (Table 1) confirm this, showing well-dispersed particles with no aggregation, which reflects the strong electrostatic repulsion between particles under the studied conditions.

**Table 1:**
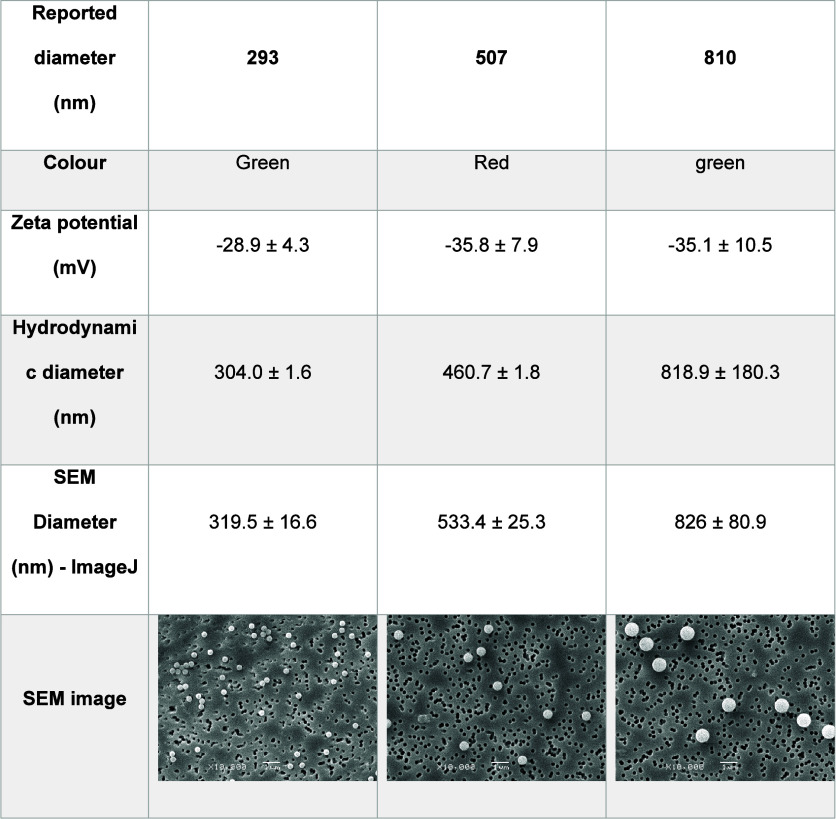
Characteristics of PS-OSO_3_^–^ NPs

Based on the NTA, the hydrodynamic diameter of the smallest nanoparticle was 304.0 ± 1.6 nm, which is slightly larger than the reported value (Table 1). The same was observed for 810 nm particles; a slightly larger hydrodynamic size of 818.9 ± 180.3 nm was recorded. This is not entirely unexpected, as the system measures the hydrodynamic size of hydrated particles. For the 507 nm particles, the hydrodynamic size was 460.7 ± 1.8 nm, which is lower than the reported value. While this is not ideal, some interactions between solvent molecules and particle surfaces could lead to more compact solvation layer, which may result in a smaller apparent hydrodynamic size.^56^ To further establish the particle sizes, ImageJ software (v2.14.0) was used to determine the average particle size of the nanoplastics based on the SEM images (SI Text S1). The reported diameters of all particles seemed to agree relatively well with the estimated diameter based on SEM imaging (Table 1). However, contrary to the expected trend, the hydrodynamic diameters of the NPs were smaller than their diameter determined using SEM. This could be due to sample preparation for SEM analysis, which included particle drying, vacuum exposure, surface coating of particles to make them conductive, and particle interaction with an electron beam. These may have caused minor flattening and deformation in the particles, increasing their apparent dimensions. Beyond this, the SEM analysis of the particles at 10,000× magnification and 50 nm resolution shows smooth, monodisperse NPs and spherical particles (Table 1).

### 3.2 Nanoplastics Quantification

#### 3.2.1 Validation of the Flow Cytometer for Nanoplastics Detection

To establish the applicability of FCM, initial tests were performed using tap water and tap water spiked with 810 nm PS NPs to concentrations of 0.2, 2, and 20 mg/L. Figure 1 shows the dot plots generated from these runs. These plots are divided into four quadrants (Q1–Q4). Particles within Q1 (upper left corner) show a high degree of scattering but low fluorescent intensity. Particles within Q2 (upper right corner) show a high degree of scattering and a high fluorescence intensity. Target particles are expected to be observed in this quadrant. Distinct cluster(s) of particles within this quadrant are gated out for further analysis. Particles within Q3 (lower right corner) show high fluorescence intensity but a low degree of scattering, while particles within Q4 (lower left corner) show both a low degree of scattering and fluorescence intensity. These particles are considered as noise.^45^

In tap water, all observed particles fall within Q4 (95.81%) and Q1 (4.19%) of the dot plot (Figure 1A). This indicates that the recorded particles within the tap water did not give off fluorescent signals strong enough to be detected as the target particles and therefore fall within the noise region. The introduction of 0.2 mg/L 810 nm PS nanoplastics (Figure 1b), equivalent to ∼6.85 × 10^8^ particles/L, leads to a clear response in Q2, with ∼13.52% of the 10,000 events being observed within this quadrant (Figure 1B). This response shows the presence of fluorescent particles, but in a low concentration relative to the observed noise in Q4 (∼76.9%). Increasing the concentration of 810 nm PS nanoplastics to 2 mg/L (∼6.85 × 10^9^ particles/L) substantially increased the overall response in Q2, with the majority of the reported events (∼79.15%) being observed within this quadrant (Figure 1C). At 20 mg/L (∼6.85 × 10^10^ particles/L), this response of particles in Q2 further increased to ∼88.79% (Figure 1D) of all events. This indicates that the PS nanoplastics generate fluorescent signals strong enough to be distinguished from background noise, allowing for clear detection in this quadrant, with signal intensity corresponding to their concentrations. Similar observations were made with real surface water (Figure S4). To ensure that the amount of spiked fluorescent particles correlated with the detected signals in quadrant 2, gradient dilutions (×1000, ×10000, ×100000) of 20 mg 810 nm NP/L were performed and analyzed (Figure 2A). On the *y*-axis, the “events” represent the number of particles detected by the equipment as the particles pass through the laser beam in the flow cell. On the *x*-axis, the “particles/μL” represents the concentration of particles in the sample flowing through the cytometer (concentration of particles that was fed) per unit volume. The correlation between spiked particles and the detected signal was established, with a high precision (*R* ^2^ = 0.99).

**Figure 2:**
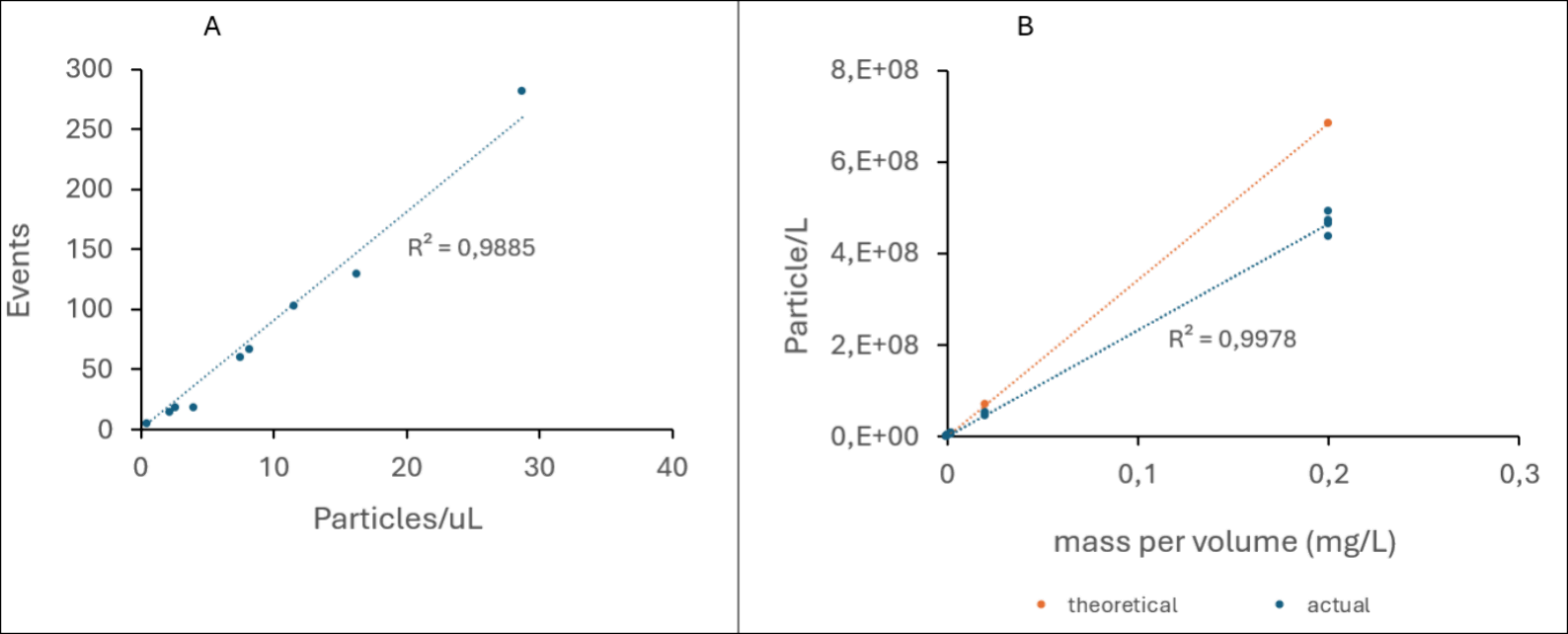
Validation of FCM for NP quantification. (A) Correlation between spiked particles and detected signals in quadrant 2. (B) correlation between mass concentration of particles vs number of particles—theoretical vs actual values.

To further validate the performance of FCM, PS NP suspensions with mass concentrations ranging from 0.2 μg/L to 0.2 mg/L were prepared and analyzed. The particle concentrations measured using FCM were compared with theoretical number concentrations, calculated based on the supplier-reported particle diameter and density (see the Supporting Information, Section SI2). As shown in Figure 2B, a strong linear correlation was observed between the mass concentration and the particle concentration measured by FCM (R^2^ ≈ 1), indicating a consistent FCM response across the tested concentration range. However, a noticeable discrepancy was observed between the measured particle concentrations and the theoretical values. This disparity may stem from overestimation in the theoretical calculations, which assume ideal conditions such as monodisperse spherical particles, uniform staining efficiency, and precise solids content. In reality, minor particle aggregation, deviations in particle shape or size, or incomplete dye labeling could contribute to the lower experimental counts observed.

#### 3.2.2 Comparing FCM and Turbidity for NP Quantification in Coagulation Experiments

The efficacy of FCM and turbidity measurements to quantify nanoplastics removal was studied at three different concentrations of 810 nm PS NPs: 0.2, 2, and 20 mg/L (Figure 3). Previous studies mainly focused on NP concentrations around 5 to 10 mg/L for applying turbidity as a measuring technique.^29,30,57^ In these studies, turbidity values were correlated to the NP concentration using calibration curves. However, the relationship between turbidity and particle concentration is complex and may not always give an accurate indication of the particle concentration within a solution.^33^ This was further corroborated by Yan,^58^ who compared the calibration curves of turbidity, ultraviolet absorbance (UV-ABS), and fluorescence spectroscopy to quantify nanoplastics and noted the lack of selectivity and sensitivity of turbidity at low NP concentrations.

At a nanoplastic concentration of 20 mg/L (Figure 3A), identical removal efficiencies with both FCM and turbidity were observed, regardless of the coagulant dose. When the nanoplastic concentration decreases to 2 mg/L, a clear deviation in the determined removal efficiency at lower coagulant dosages was observed (Figure 3B). At coagulant doses of 0 and 2 mg Fe^3+^/L, FCM observed higher removal efficiencies of ∼15 and ∼60%, than turbidity, with efficiencies of ∼−3 and ∼15%, respectively. This effect was even more pronounced at a NP concentration of 0.2 mg/L, where the FCM observed better nanoplastic removal efficiencies as compared to the turbidity measurements (Table S3), regardless of coagulant dose (Figure 3C). The negative removal (−3%) in turbidity may be attributed to nonuniform particle distribution within the sample at low particle concentrations before and after the coagulation treatment, as will be described in the upcoming section. Further details are provided in the following section.

The observed trend of decreasing comparability between the removal efficiencies of FCM and the turbidity can be explained by the operating principles of the two measuring techniques. Turbidity provides a qualitative analysis of particles in the suspension. The process is dependent on the scattering of light by suspended particles in solution. The higher the concentration and size of particles in suspension, the higher the intensity of light scattered, leading to higher turbidity.^59,60^ Consequently, turbidity measurements are limited by the concentration and size of the suspended particles in the sample.

At low particle concentrations, a nonuniform distribution of particles within the suspension is more likely. It follows that areas of high particle concentrations will give rise to higher turbidity readings, while areas of low particle concentrations will result in low turbidity readings, leading to inconsistent and inaccurate results.^60,61^ In contrast, FCM, being a high-resolution single-particle analytical technique, analyzes particles based on events. Therefore, higher removal efficiencies, consistent with the coagulant dose, could be determined even at lower NP concentrations (Figure 3C). Foladori^41^ applied FCM to quantify the removal of 0.55 mg/L of 1 μm PS NPs from raw and presettled wastewater after coagulation/flocculation and sedimentation using 12 mg Al^3+^/L. The authors reported a maximum removal efficiency of 97%, which is consistent with our observations in Figure 3C where a maximum removal efficiency of ∼ 98% was possible to achieve for 0.2 mg/L 810 nm PS NPs using FCM.

At low NPs concentrations, the contribution of the dosed Fe^3+^ to the observed turbidity becomes visible. The production of soluble iron hydroxo-complexes such as [Fe­(H2O)_4_(OH)_2_]^+^ and [Fe_3_(H2O)_8_(OH)_4_]^5+^ during hydrolysis of FeCl_3_ coagulant leads to a characteristic yellow to brown coloration that can absorb light, hence reducing the resulting scattered and detected light. Consequently, the final turbidity is affected.^62^ This may explain the observation in Figure 3C. Fluorescence-based techniques, such as FCM, possess the ability to distinguish between noise and target particles, and as such, interferences from the coagulant or other background noises are avoided. Even though turbidity removal has been widely used as an indicator of NP removal, the results shown demonstrate that the extent of the application of turbidity removal for NP removal is limited.

### 3.3 Effects of Fe^3+^ Dose

The effects of coagulant dose on the removal of NPs were studied by varying the coagulant dose from 0 to 30 mg Fe^3+^/L at the three concentrations of NPs (20, 2, and 0.2 mg NP/L). At 20 mg NP/L (Figure 3A), a general trend of increasing NP removal efficiency with increasing Fe^3+^ dose was observed until 10 mg Fe^3+/^L, beyond which the removal efficiency plateaued at ∼95%. Similar observations were made in Figure 3B (2 mg of NP/L), except that at 0 mg of Fe^3+^/L dose (without the addition of coagulant), ∼15% NP removal was observed. Similar to Figure 3B, in Figure 3C, a removal of ∼30% of the NP was observed at a 0 mg Fe^3+^/L dose. Interestingly, at a coagulant dose of 2 mg Fe^3+^/L, no removal was observed for NPs, which was unexpected (Figure 3C). At 5 mg of Fe^3+^/L dose, ∼85% removal efficiency was observed. Beyond that, removal efficiency plateaued at ∼98% for coagulant doses of 10, 20, and 30 mg Fe^3+^/L.

**Figure 3:**
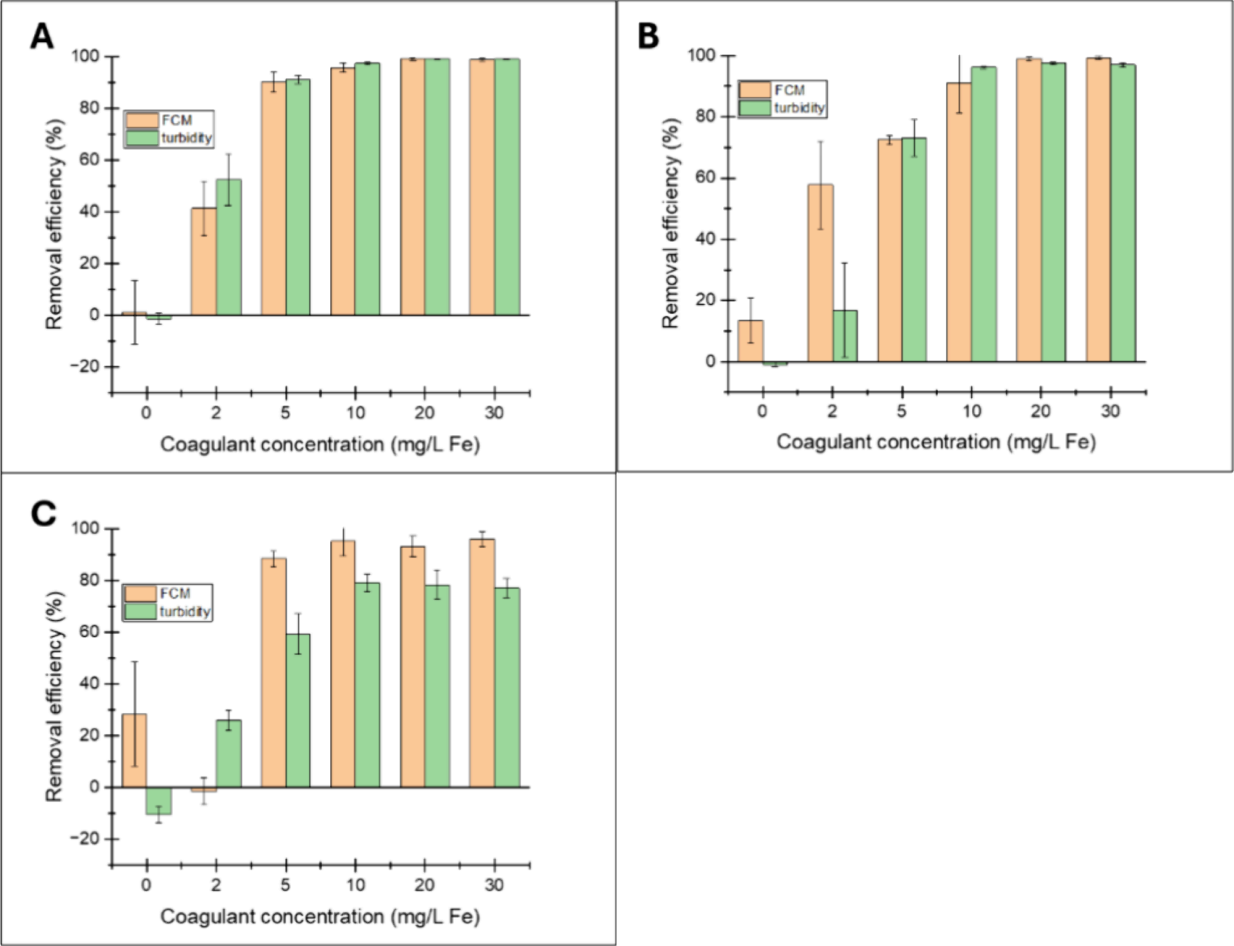
Removal efficiencies of 810 nm PS NPs at different NP concentrations, as measured by FCM and turbidity. (A) 20 mg/L 810 nm PS NPs; (B) 2 mg/L 810 nm PS NPs; and (C) 0.2 mg/L 810 nm PS NPs. Error bars represent the standard deviation of three measurements.

The observed increase in NP removal efficiency can be attributed to the increased coagulant dose, as an increase in coagulant dose implies the presence of more Fe-hydrolysates to neutralize the negative surface charges (-OSO_3_^–^) of the NPs in the solution. Additionally, with increased Fe-dose, there is an increased frequency of collision between the PS NPs and the Fe-hydrolysates, consequently enhancing the aggregation of the particles.^17^ Li, Zhang^57^ made similar observations, reporting an increase in removal efficiency of 500 nm PS NPs from 94.3% to 97.6% when the coagulant dose was increased from 5.6 mg Fe^3+^/L to 28 mg Fe^3+^/L. Comparable observations have been reported by previous research.^25,63,64^

From Figure 3B,C, the recorded removal of NPs (∼15 and ∼30%, respectively) at 0 mg Fe^3+^/L dose may be due to the combined effects of the composition of the tap water (SI Table S1), mixing regimes, and the density of PS NPs (1.05 g/cm^3^), which may have promoted some aggregation and settling of the particle. Previous work by Zhou^64^ reported up to 51% removal of PS NPs after a jar test experiment without the addition of coagulants. The absence of measurable NP removal at a dosage of 2 mg Fe^3+^/L (Figure 3C) may be attributed to the limited frequency of collisions between the few particles and coagulant species, resulting in inefficient destabilization and slower, incomplete aggregation.^65^

The observed plateau at 10, 20, and 30 mg Fe^3+^/L dose in all conditions (Figure 3) may be due to the system reaching its maximum aggregation capacity. At this point, further addition of coagulant does not end in further destabilization or aggregation, as the potential binding sites on the NPs are occupied and the system is saturated.^19^ Additionally, this could be because the NP removal approached 100%. This may explain the observed trend and points to an optimum coagulant dose range of 5–10 mg Fe^3+^/L for the studied conditions.

### 3.4 Coagulation Mechanism

It has long been known that the removal of particles through coagulation and flocculation occurs through two main mechanisms, namely, charge neutralization and sweep coagulation.^19^ The predominant mechanisms are mainly dependent on the pH of the solution, the coagulant dose, and the coagulant type.^66^ To get an idea of the coagulation mechanism dominant during the coagulation/flocculation of the PS NPs, SEM analysis was performed on the ferric hydroxide precipitates after coagulation, flocculation, and settling (Figure 4). In Figure 4A, the SEM image of 2 mg/L of 810 nm PS NPs treated with 10 mg of Fe^3+^/L shows that the NPs are enmeshed within the hydroxide precipitates. This is typical for the sweep coagulation mechanism, where there is the formation of a combination of small polynuclear hydrolysis species such as Fe_2_(OH)_2_^4+^ and Fe_3_(OH)_4_^5+^, large polymeric species such as Fe_*n*_(OH)_*m*_(H_2_O)_*x*_^(3*n*–*m*)^ or Fe_*x*_O_*y*_(OH)_*z*_^(3*x*–2*y*–*z*)+^, as well as amorphous ferric hydroxide (Fe­(OH)_3_ am) due to the relatively high concentration of FeCl_3_ dose and favorable pH of 6–8.^67,68^ Within these conditions of FeCl_3_ dosage and pH, the hydrolysis species precipitate out of solution, providing enough surface area for adsorption and bridging effects, consequently capturing the NPs and removing them from suspension.^19^ At the mentioned conditions of 2 mg/L nanoparticles, a coagulant dose of 10 mg Fe^3+^/L, and a pH range of 7.3–7.9, the ratio of coagulant to PS NPs and favorable pH led to the right conditions for sweep coagulation (Figure 4A).

**Figure 4:**
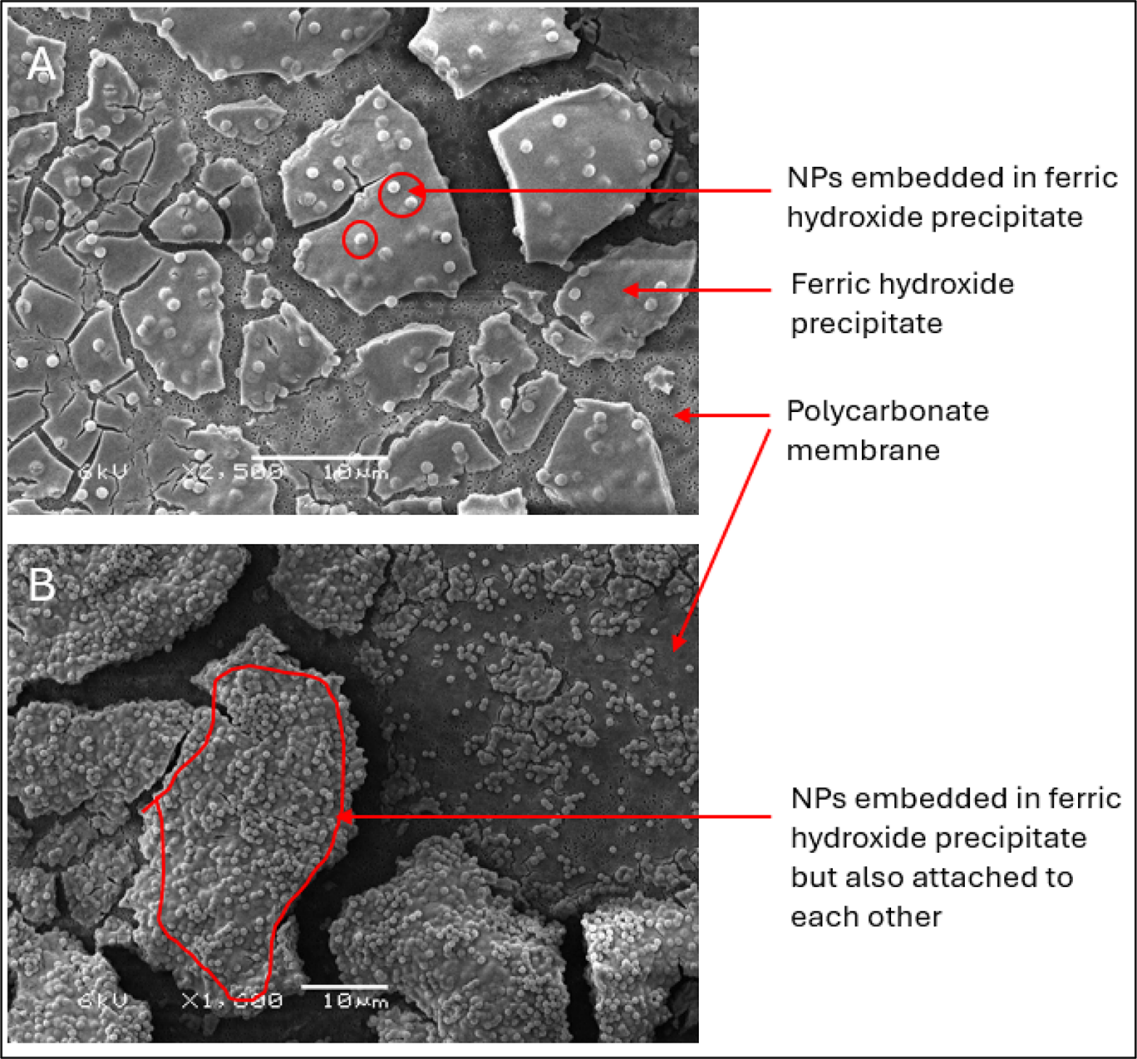
SEM images of precipitates of ferric hydroxide containing PS NPs. (A) Individual NPs embedded in 2 mg/L 810 nm PS NPs + 10 mg Fe^3+^/L; (B) aggregated NPs embedded in 20 mg Fe^3+^/L 810 nm PS NPs + 10 mg Fe^3+^/L.

In Figure 4B, the SEM image of 20 mg/L 810 nm PS NPs treated with 10 mg of Fe^3+^/L shows a clear change in the coagulation mechanism. Unlike the previously described condition, the NPs appear to be attached to each other and also enmeshed within the ferric hydroxide flocs. Quite clearly, multiple coagulation mechanisms are at play, which could predominantly be (1) charge neutralization and (2) sweep coagulation.^69^ With charge neutralization, the positively charged Fe-hydrolysate, such as Fe^3+^ and Fe­(OH)^2+^, is adsorbed onto the surface of the negatively charged PS NPs to neutralize them and compress their double layer, causing NPs to become unstable, aggregate, and create flocs.^20^ A major basis for charge neutralization, as suggested by Edzwald,^70^ is the presence of a low coagulant concentration relative to the particle concentration, which was observed in this case. Beyond charge neutralization, sweep coagulation, as described in the previous paragraph, is also possible, as evidenced by the enmeshment of NPs in the Fe-hydroxide precipitates. The aggregation of particles during coagulation through multiple mechanisms is not uncommon. Charge neutralization is noted to usually precede sweep coagulation, and for a system with a low coagulant dose relative to particle concentration, charge neutralization is expected to be the more dominant mechanism.^67,71^ These observations provide some insight into the coagulation mechanisms during the test.

### 3.5 Effects of Slow Mixing Speed

Enhanced coagulation approaches involve, among other things, using coagulation aids, improving the hydrodynamic conditions, changing pH, or increasing the coagulant dose.^72^ Our approach explored improving the hydrodynamic conditions by keeping the rapid mixing speed constant at 300 rpm (*G*-value = 632 s^–1^) and varying the slow mixing speed during flocculation at three different Fe-doses (Figure 5A).

**Figure 5:**
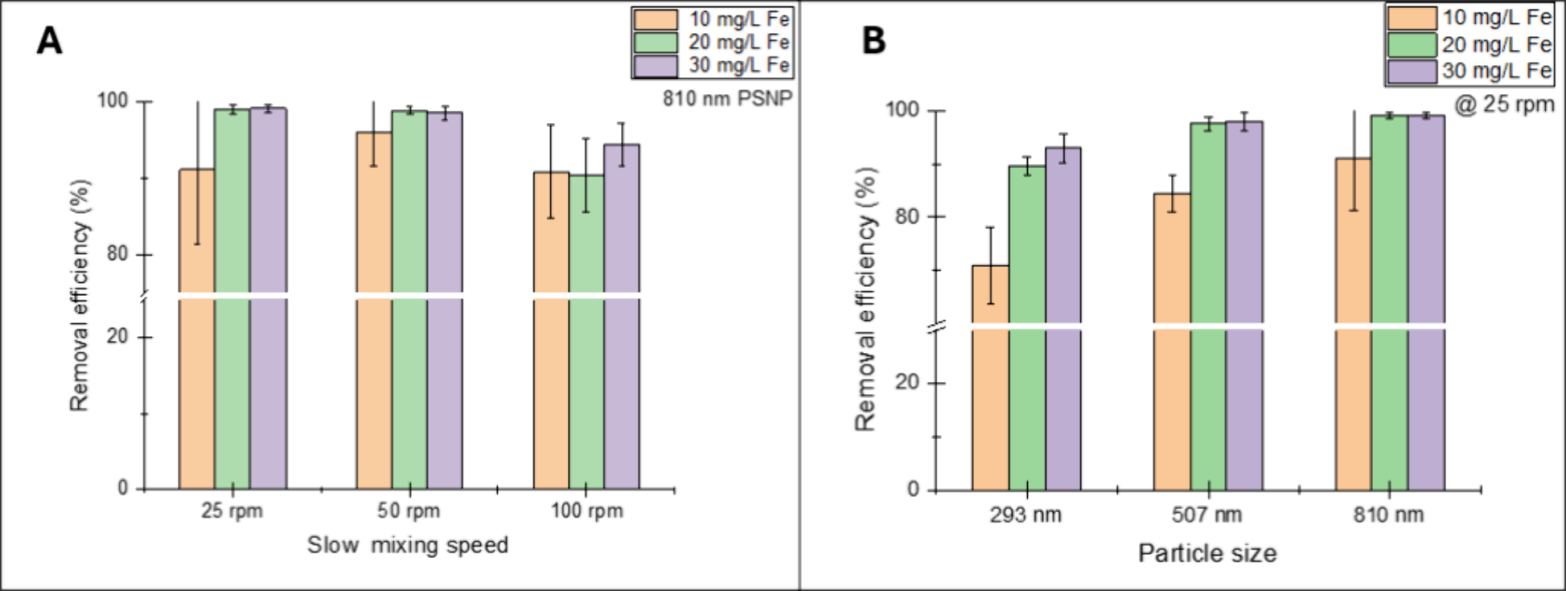
(A) Effects of slow mixing speed on the removal efficiency of 810 nm PS NPs through coagulation/flocculation; (B) effects particle size on the removal efficiency of PS NPs through coagulation/flocculation at 25 rpm. Error bars represent standard deviation of three measurements.

From the figure, near identical removal efficiencies (∼99%) were observed for 25 (14 s^–1^) and 50 rpm (40 s^–1^) at coagulant dosages of 20 and 30 mg of Fe^3+^/L. At a lower coagulant dose of 10 mg of Fe^3+^/L, a slight difference in removal efficiencies (∼91% at 25 rpm and ∼95% at 50 rpm) was observed. For the highest investigated mixing speed of 100 rpm (125 s^–1^), similar removal efficiencies (∼90%) were observed for 10 and 20 mg Fe^3+^/L dose, while a minor increase in removal to ∼95% was observed for the 30 mg Fe^3+^/L dose.

Slow mixing during flocculation is necessary for floc growth. During this process, microflocs formed during coagulation (rapid mixing) collide with each other to form larger aggregates that can settle more efficiently. An optimum range of slow mixing speed should promote floc growth without breaking it.^73^ The observed effects of the slow mixing speeds on NP removal efficiency may be explained by the fact that at 25 and 50 rpm, enough relative velocity gradient was induced between particles to enhance collision, growth, and subsequent settling of the flocs,^19^ which led to removal efficiencies exceeding 95%. On the other hand, while the NP removal efficiency at 100 rpm was good (90–95%), the observed differences compared to 25 and 50 rpm may be due to the breakage of flocs caused by the higher relative velocity gradient induced by mixing at 100 rpm. This may have led to less settleable flocs and consequently lower removal efficiencies. Similar findings have been documented by Li, Busquets,^38^ who observed a decline in the removal of PS beads when the slow mixing speed was increased from 50 rpm (23 s^–1^) to 100 rpm (66 s^–1^). As one of the main objectives of this study, the above results prove that excellent removal efficiency can be achieved with a slow mixing speed of 25 rpm (14 s^–1^).

### 3.6 Effects of Particle Size

To establish the effect of particle size on NP removal, experiments were conducted using three PS NPs with sizes of 293, 507, and 810 nm, at coagulant doses of 10, 20, and 30 mg Fe^3+^/L. A clear increase in removal efficiency with increasing particle size, regardless of the ferric chloride dose, was observed (Figure 5B). In line with expectations, the lowest removal of each particle size was observed at 10 mg Fe^3+^/L, with removals of ∼70, ∼85, and ∼90%, for 293, 507, and 810 nm, respectively. At the coagulant doses of 20 and 30 mg of Fe^3+^/L, the difference in the removal of 507 and 810 nm PS NPs became negligible, with removal efficiencies of ∼99% (Figure 5B). In contrast, the removal efficiency of the smallest studied particle (293 nm) showed a dependency on Fe-dose, increasing from ∼70% at 10 mg/L Fe-dose to ∼93% at 30 mg of Fe^3+^/L.

Particle size plays a significant role in the efficiency of particle collision during the coagulation, flocculation, and settling of suspended and colloidal particles in water. It could be seen that the smallest NPs have a lower degree of removal efficiency compared to the larger particles, despite their lower zeta potential (see Table 1), which suggests reduced stability against aggregation. Still, the effects of smaller particle size appear to more than compensate for the lower zeta potential. Additionally, large particles have a higher tendency to collide with coagulants, form larger aggregates, and settle more easily under the influence of gravity compared to small particles.^62^ This effect was observed with the 507 and 810 nm PS NPs (Figure 5B), which show a removal efficiency higher than that of 293 nm PS NPs. Within the same range of particle sizes, Zhang^17^ observed a similar trend in the removal efficiency of PS NPs. The authors noted a removal efficiency of 83.6% of PS NPs with sizes of 500 and 1000 nm after coagulation/flocculation and settling, while the smaller size particles (100 nm) were poorly removed with an efficiency of 55.4%. These observations are further corroborated by the observations of Li,^57^ who compared the coagulation performance of five commonly used coagulants (polyacrylamide, polyaluminum sulfate, aluminum sulfate (Al^3+^), polyferric sulfate (poly-Fe), and ferric sulfate (Fe^3+^)) in the removal of 100 and 500 nm PS NPs. The authors reported removal efficiencies of 83.15 and 82.5% for 500 nm PS NPs using 0.5 mM poly-Fe (6 mg Fe^3+^/L) and 1 mM Fe^3+^ (55.8 mg Fe^3+^/L), respectively. This was the highest removal compared to the rest. For the 100 nm PS NPs, a poor removal efficiency was recorded in all cases.

The observed increasing removal efficiency with increasing Fe-dose for the 293 nm particles (Figure 5B) was expected. Small particles have a large surface area-to-volume ratio in solution, which corresponds to an overall higher surface charge density.^62^ This implies stronger electrostatic repulsion between particles, making them more stable. By introducing more coagulants, enough Fe-hydrolysates, i.e., counterions, are released into the solution to neutralize the charges, form more flocs, and promote aggregation.^74^ This could explain the increase in removal efficiency from ∼70% at a 10 mg/L Fe-dose to ∼93% at 30 mg Fe^3+^/L.

### 3.7 NP Removal from Real Surface Water

The applicability of FCM to detect NPs under environmental conditions was evaluated using real surface water collected from a river in Leeuwarden, Friesland, The Netherlands. The river water samples were spiked with 810 nm polystyrene nanoplastics (PS NPs) at concentrations of 0.2, 2, and 20 mg/L. Coagulation experiments were conducted at a slow mixing speed of 25 rpm, with a fixed coagulant dose of 10 mg Fe^3+^/L. At the lowest concentration (0.2 mg NP/L), nearly complete removal of NPs was achieved (∼100% removal, Figure 6). For the higher concentrations (2 and 20 mg NP/L), lower removal efficiencies of ∼85% were observed.

**Figure 6:**
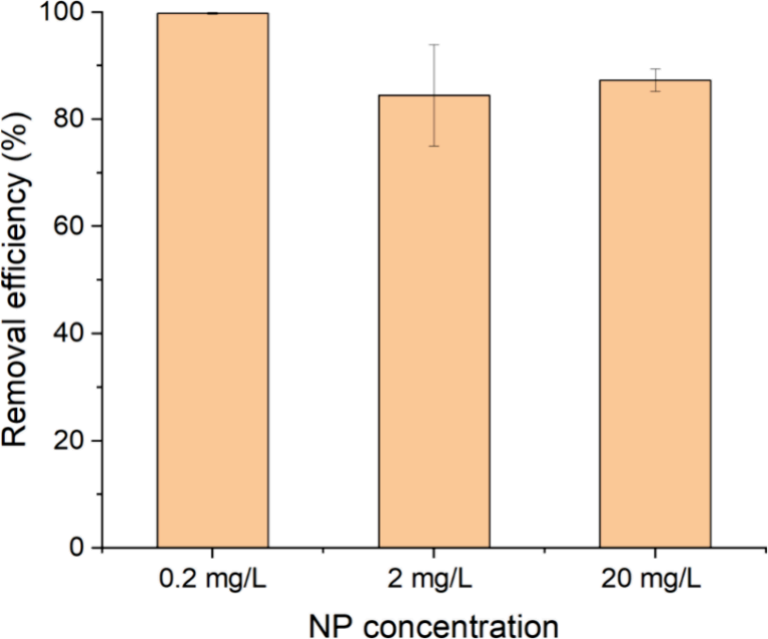
Removal efficiencies of 810 nm PS NPs from real surface water as measured by FCM. Error bars represent the standard deviation of three measurements.

The high removal efficiency of NPs at 0.2 mg of NP/L is in line with expectations. At this low nanoplastic concentration, a coagulant dose of 10 mg of Fe^3+^/L provided an excess of Fe-hydrolysis products in solution, which facilitated effective nanoparticle removal primarily through sweep flocculation. Under identical conditions, a similar removal efficiency (∼100%) was observed in tap water, indicating that at low NP concentrations, the differences in the water matrix had minimal influence on removal performance. In contrast, at higher NP concentrations (2 and 20 mg NP/L), the removal efficiency in river water decreased to approximately 85%, whereas in tap water, it remained higher at around 96%. This discrepancy is likely due to differences in the physicochemical properties of the two water sources. As shown in Tables S1 and S2, the ionic strength of tap water (4.45 mM) is substantially higher than that of river water (0.013 mM). The higher the ionic strength, the more prone the particles are to aggregation due to compression of the electric double layer surrounding the particles, reducing electrostatic repulsion and subsequently leading to their removal.^75^ Additionally, the river water contained a significantly higher concentration of dissolved organic carbon (DOC; ∼17.6 mg/L) compared to tap water (∼2.97 mg/L). High DOC levels can enhance colloidal stability by providing steric stabilization, which hinders aggregation and may account for the lower NP removal efficiency observed in river water.^76^ In contrast, the lower DOC concentration in tap water exerts less of a stabilizing influence, allowing for more effective aggregation and removal of nanoparticles under the same coagulation conditions.

### 3.8 Impact and Outlook

Nanoplastics pose two significant challenges due to their small size and widespread presence: (1) their accurate quantification and (2) their efficient removal using conventional water treatment methods. This study offers preliminary insights by applying a fluorescence-based quantification method (FCM) to measure the removal of fluorescent polystyrene nanoplastics (PS NPs) during enhanced coagulation/flocculation processes. The FCM method demonstrated greater accuracy and consistency than traditional turbidity measurements for quantifying nanoplastics, which holds promise for advancing precise NP detection in water treatment plants. Moreover, the study highlights that enhanced coagulation conditions, particularly with improved slow mixing, can achieve excellent NP removal. This suggests that existing water treatment facilities could enhance coagulation-flocculation processes by adjusting mixing conditions and the used coagulant dose to improve their NP removal efficiency. However, since this research was conducted mainly using tap water, future studies should explore more complex water matrices, such as those containing natural organic matter like humic acids or clay particles, in detail. This represents the next phase of our research. This would help validate the effectiveness of fluorescence-based FCM in environments that more closely resemble real-world water treatment scenarios. Additionally, future studies should explore the potential of flow cytometry (FCM) to provide size distribution data, which would allow for the simultaneous determination of both the particle concentration and size. This would further enhance the utility of FCM in nanoplastic analysis.

## 4 Conclusions

This study aimed to show the applicability of FCM to accurately quantify fluorescent PS NPs during enhanced coagulation. The fluorescence-based technique was compared with the commonly used turbidity measurements for NP quantification. FCM proved to be more accurate than turbidity measurements, particularly at lower NP concentrations. At high NP concentrations (2–20 mg/L) turbidity showed comparable NP removal efficiency with FCM. However, at low NP concentrations (0.2 mg/L), turbidity measurements were no longer comparable to FCM, implying the limitation of turbidity to accurately quantify NPs at low concentrations. The research also identified key factors influencing NP removal, such as FeCl_3_ dosage (optimal dose of 5–10 mg/L) and optimal slow mixing speed of 25 rpm (14 s^–1^), which enhanced the coagulation/flocculation efficiency. Furthermore, the study highlighted the impact of particle size on removal efficiency, with smaller particles showing a stronger dependence on the coagulant dosage than larger particles. Overall, findings from this study provide valuable insights for improving coagulation/flocculation toward NP removal from water and provide some basis for further research and technology development toward NP removal from water and its rapid quantification.

## Supplementary Material



## Notes

Additional experimental details on composition of the tap water used, image analysis, arrangement of samples for flow cytometry analysis, and an additional figure that supports the results (PDF)
