# Supplementary material for: Evaluating the Efficiency of Enhanced Coagulation for Nanoplastics Removal Using Flow Cytometry
Source: ACS ES T Water. 2025 Jun 11;5(7):3908–19. doi: 10.1021/acsestwater.5c00219 (PMC12261319; doi:10.1021/acsestwater.5c00219)
Supplement: Supplementary file 1 [file ew5c00219_si_001.pdf]

Evaluating the Efficiency of Enhanced Coagulation for Nanoplastics Removal Using Flow Cytometry

Elorm Obotey Ezugbe[1,2,] Samuel Benjamin Rutten[2], Bianca de Vries-Onclin[2], R. Martijn Wagterveld[2], Wiebe de Vos[1], and Saskia Lindhoud[3]*

[1]Membrane Science and Technology, University of Twente, Drienerlolaan 5, 7522 NB Enschede, The Netherlands

[2]Wetsus, European Centre of Excellence for Sustainable Water Technology, Oostergoweg 9, 8911 MA Leeuwarden, The Netherlands

[3]Department of Molecules & Materials, University of Twente,7522NB Enschede, The Netherlands

* Corresponding Author: s.lindhoud@utwente.nl

Table S1: Composition of tap water

Table S2: Composition of river water (Potmarge)

Table S3: Turbidity of supernatant samples

Table S4: average diameter of 10 randomly selected particles analyzed using ImageJ software

Figure S1: Vacuum filtration of real surface water

Figure S2: Arrangement of samples on a 96-well plate.

Figure S3: Cytogram of Suwannee River III humic acids.

Figure S4: Cytogram of real surface water spiked with different concentrations of NPs.

Table S1: Composition of tap water

Parameter	Value (mg/L)
**Cl**	39.1
**NO_2**	<0.10
**NO_3**	10.1
**PO_4**	<0.10
**SO_4**	0.36
**Total Carbon**	55.1
***NPOC**	2.97
**Inorganic carbon (IC)**	52.1
**Ca**	31.6
**Cu**	0.6

**Fe**	0.004
**K**	2.0
**Mg**	8.9
**Na**	68.3

**NPOC: non purgeable organic carbon*

Table S2: Composition of Potmarge

Parameter	Value (mg/L)
**Calcium (IC)**	0,108
**Magnesium (IC)**	0,0248
**Potassium (IC)**	0,0111
**Sodium (IC)**	0,0916
**Chloride**	0,156
**Nitrate**	0,006
**Nitrite**	0,00553
**Phosphate**	0,001
**Sulphate**	0,0608
**DOC***	17.6

*DOC = dissolved organic carbon

Table S3: Turbidity of supernatants at different coagulant doses.

Turbidity of supernatant (NTU)			
	concentration of NP (mg/L 810 nm PS NP)		
mg/L Fe^{3+}	0.2	2	20

0	1,06 ± 0,04	9,42 ± 0,06	92,37 ± 1,24
2	0,78 ± 0,04	7,55 ± 1,40	46,32 ± 9,69
5	0,39 ± 0,07	2,49 ± 0,54	8,73 ± 1,56
10	0,22 ± 0,04	0,36 ± 0,04	2,42 + 0,33
20	0,21 ± 0,05	0,23 ± 0,03	0,91 ± 0,11
30	0,22 ± 0,04	0,29 ± 0,07	0,93 ± 0,24

Text S1: Procedure for Image analysis ImageJ software (version 2.14.0)

SEM images were loaded into the ImageJ software.

To set the scale to the appropriate unit, the following settings were used:

- Distance in pixels = 100.00 (obtained by measuring the scale bar on the SEM image)
- Known distance = 1.00 (representing length of the scale bar in μm).
- Pixel aspect ratio = 1.0
- Unit of length = μm

These settings brought the scale to 100 pixels/unit

To obtain the diameter of the spherical particles, a straight line was drawn across the hight/length of the particles and the values recorded.

This was repeated for 10 random particles as shown in the table below.

Table S4: average diameter of 10 randomly selected particles analysed using ImageJ software

**NP**	**293 nm**	**507 nm**	**810 nm**
	**diameter (nm)**		
**1**	321	532	807
**2**	321	530	809
**3**	360	538	800

**4**	310	545	736
**5**	300	517	819
**6**	322	506	798
**7**	310	520	772
**8**	311	541	870
**9**	330	510	817
**10**	310	595	1035
**avg**	319.5	533.4	826.3
**std dev**	16.61	25.32	80.88

Filtration of Potmarge samples before experiments

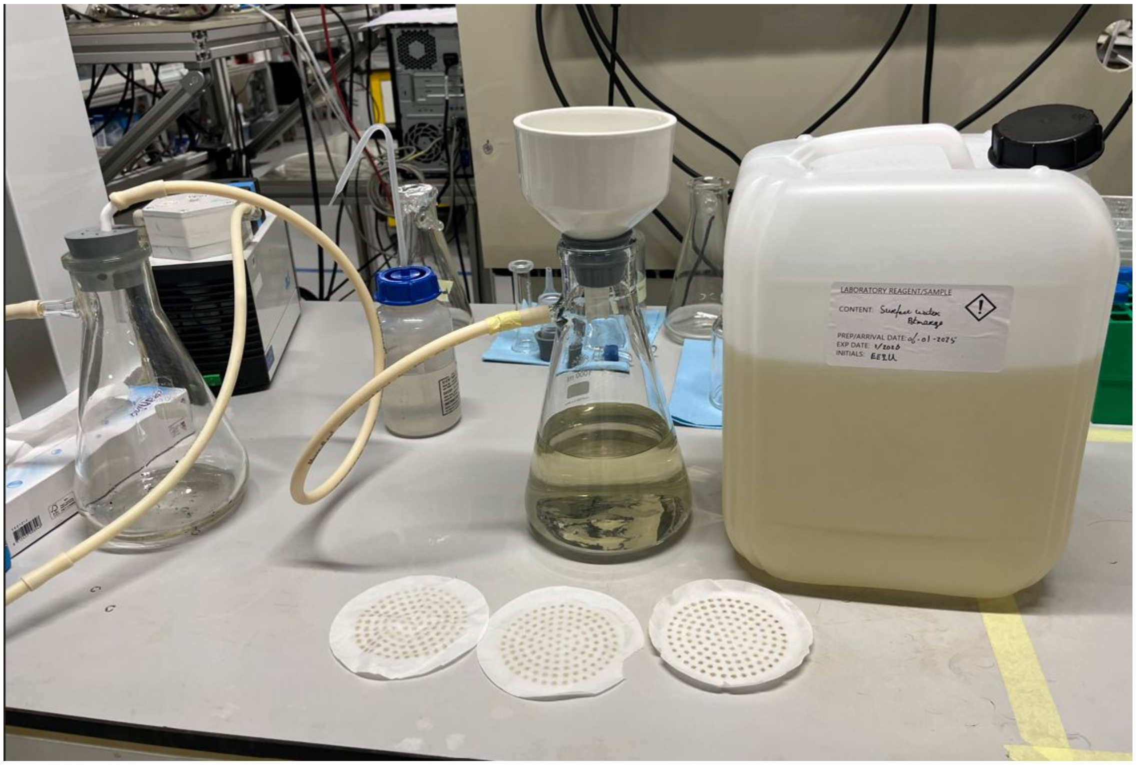

Figure S1: vacuum filtration of Potmarge samples using 12-15 µm filter paper. Samples collected on the 6th of January 2025

Sample arrangements on 96-well plate for FCM analysis

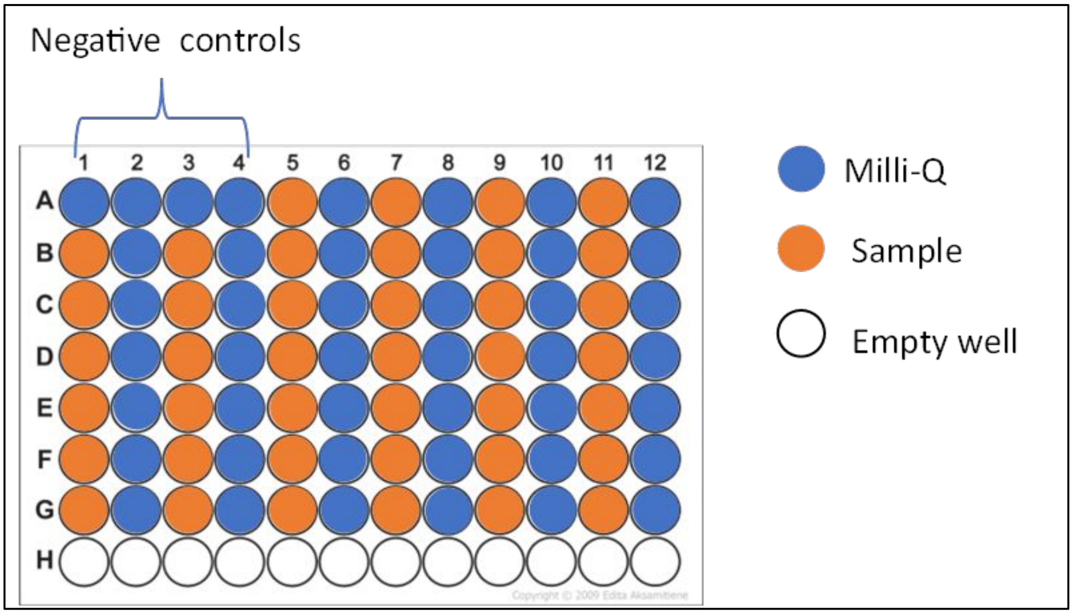

Figure S2: Typical arrangement of samples on a 96-well plate.

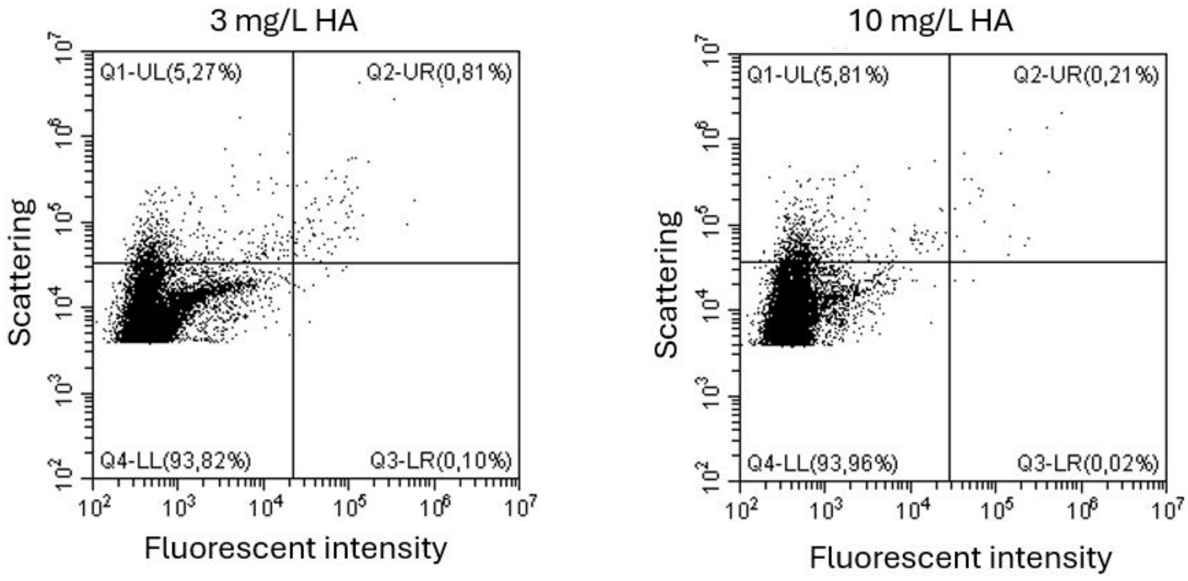

Figure S3: Cytogram of humic acids. A) 3 mg/L and B) 10 mg/L

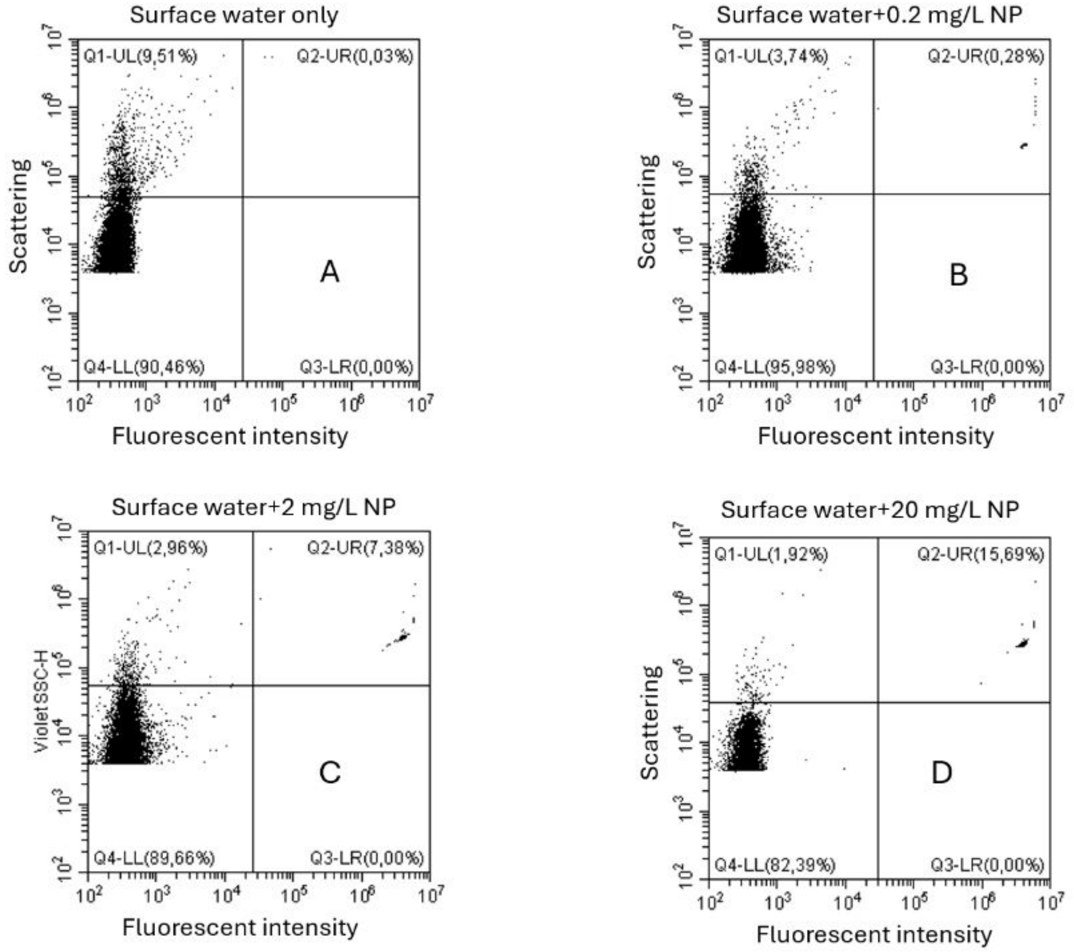

Figure S4: Dot plots of 810 nm PS NPs generated from FCM. A) raw real surface water; B) 0.2 mg/L NP; C) 2 mg/L; D) 20 mg/L.
